# Characteristic and Early Discontinuation of Obsessive-Compulsive Disorder Trials Registered on ClinicalTrials.gov

**DOI:** 10.3389/fpsyt.2021.650057

**Published:** 2021-07-26

**Authors:** Shanxia Luo, Qiong Guo, Liu Yang, Yifan Cheng, Youlin Long, Xinyi Wang, Liqin Liu, Zixin Yang, Tengyue Hu, Liang Du, Min Chen, Ga Liao

**Affiliations:** ^1^Department of Mental Health Center, West China Hospital of Sichuan University, Chengdu, China; ^2^Chinese Evidence-Based Medicine Center, West China Hospital, Sichuan University, Chengdu, China; ^3^Medical Device Regulatory Research and Evaluation Centre, West China Hospital, Sichuan University, Chengdu, China; ^4^West China School of Medicine, Sichuan University, Chengdu, China; ^5^Department of Traditional Chinese Medicine, The Third People's Hospital of Chengdu, Chengdu, China; ^6^State Key Laboratory of Oral Diseases, National Clinical Research Center for Oral Diseases, West China Hospital of Stomatology, Sichuan University, Chengdu, China

**Keywords:** obsessive-compulsive disorder, clinicaltrials.gov, early discontinuation, underreporting, delayed reporting

## Abstract

**Objective:** This study aimed to analyze the characteristics and reasons of early discontinuation of obsessive-compulsive disorder (OCD) trials registered on ClinicalTrials.gov.

**Methods:** OCD trials and relevant publications were searched on ClinicalTrials.gov and PubMed, respectively. The characteristics and details regarding the timely publication of trials were recorded. Cox regression analysis was used to explore factors associated with the early discontinuation of OCD trials.

**Results:** The analysis included 298 OCD therapy trials. Most investigations recruited <100 patients and were more likely to involve adults. Of all OCD studies identified, 67.8% were randomized and 61.4% were blind (single- or double-blind). Universities and hospitals were recorded as the two primary locations in the majority of trials. A total of 155 trials (52%) were completed; however, only 29% of those were published. Of the published trials, >70% were published at least 1 year after completion. Behavioral therapy trials were the most common type of major treatment-aimed OCD trials (39%), followed by drug trials (35.1%) and device/procedure trials (24.7%). The univariate Cox regression analysis indicated that drug trials [hazard ratio (HR) = 2.56, 95% confidence interval (CI): 1.21–5.43], absence of collaborators (HR = 3.87, 95% CI: 1.62–9.26), and sponsorship by industry (HR = 3.97, 95% CI: 1.49–10.53) were risk factors for early discontinuation of OCD trials. Further multivariate Cox regression showed that drug trials (HR = 3.93, 95% CI: 1.71–9.08) and absence of collaborators (HR = 5.17, 95% CI: 1.97–13.54) were independent risk factors for early trial discontinuation of OCD trials. The sensitivity analysis confirmed these results. Non-drug trials (OR = 3.32, 95% CI: 1.21–9.11), absence of collaborators (OR = 3.25, 95% CI: 1.10–9.60), and non-blinded trials (OR = 5.23, 95% CI: 1.05–26.2) were independent risk factors for unreported results in registry.

**Conclusion:** The diagnosis and prevention of OCD are rarely investigated in trials. Underreporting and delayed reporting remain major problems. The type of intervention and participation of collaborators are associated with early discontinuation of OCD trials.

## Introduction

Obsessive-compulsive disorder (OCD) ranks fourth among the most common mental disorders. It is characterized by the occurrence of obsessions, compulsions, or both (1). Obsessions are unwished doubts, thoughts, images, and urges that repeatedly occur in the mind of an individual. Compulsions are defined as repeated behaviors or mental acts that patients wish to perform in response to obsessions (2). The prevalence of OCD throughout the lifetime of an individual is 1.6% and varies significantly with age (3).

Although OCD occurs in all ages, it is particularly common in children and young individuals. There is no difference in the prevalence of OCD between the sexes. Females with OCD mostly exhibit compulsive behaviors (e.g., washing of hands), while males tend to demonstrate obsessions of a sexual nature (1). Individuals with OCD are less likely to be married or employed and more likely to fear contamination, which leads to undue washing (4). The World Health Organization has ranked OCD as one of the most handicapping clinical conditions due to loss of income and a decrease in quality of life (QoL) (1). The currently available literature suggests that the mean QoL of patients with OCD is lower than that of the healthy population. Thus, OCD has a marked impact on QoL (3).

Studies have shown that the treatment of OCD can be divided into three aspects: psychological, pharmacological, and surgical or neuromodulatory (5). In psychological treatment, the most effective approach is cognitive behavior therapy, particularly exposure and response prevention (6). Previous studies revealed that cognitive behavior therapy substantially reduces compulsions and obsessive thoughts of patients; nevertheless, 50% of patients are resistant to such treatment due to the lack of motivation and engagement (7). A meta-analysis yielded a mean effect size of 0.91 across 18 randomized controlled trials of treatment with serotonin reuptake inhibitors, showing a large effect (4). However, some studies reported residual symptoms after adequate treatment and relapse after discontinuation (8). Operative treatment is applied to patients with severe, treatment-resistant conditions; however, the safety and effectiveness of this treatment modality remain unknown. Other interventions include deep brain stimulation. Research on this therapeutic approach is currently ongoing, and its safety and effectiveness are under investigation (6).

ClinicalTrials.gov is the largest clinical trial registry worldwide, sharing information on clinical research (9). A large body of evidence in ClinicalTrials.gov indicates that there is a tendency toward bias in mental health trials, due to the clinician-judged or self-reported data (10), as well as study designs that often involve small sample sizes, open-label investigations, or placebo comparators (9). Furthermore, previous studies have found that the publication rate of registered research studies is low, and numerous investigations are not published for several years after completion (particularly those with null findings) (10). Moreover, only 29.9% of the completed research studies report their results to the registry. Occasionally, this underreporting of results is attributed to influence by academic medical center/hospital/other funders (9). According to the available data, 131 OCD trials were registered in ClinicalTrials.gov between 2007 and 2018 (9). However, limited attention has been paid to the characteristics of OCD trials in ClinicalTrials.gov.

The aim of this study was to provide an overview of current OCD clinical trials registered in ClinicalTrials.gov. The present analysis sought to investigate the OCD trial features, namely, the year of registry, trial phase, number of sites, funder, location of study procedures (sponsor), study status, intervention, and result reporting. Moreover, trial characteristics that may be associated with early trial discontinuation were evaluated.

## Materials and Methods

### Data Source

We searched ClinicalTrials.gov in the “condition or disease” field using the terms “Obsessive Compulsive Disorder,” “Compulsive Disorder,” and “OCD.” A dataset of OCD clinical trials registered in ClinicalTrials.gov was downloaded on May 7, 2020. Details of terms definitions are available at https://www.clinicaltrials.gov/ct2/about-studies/glossary.

### Study Selection

The OCD clinical trial dataset was restricted to interventional studies registered up to May 7, 2020. Interventional studies are defined as clinical trials in which participants are assigned to groups that receive or do not receive intervention. We only included studies of OCD and OCD combined with other diseases. Studies in which OCD was only one of the symptoms of diseases, such as anxiety disorder, depressive disorder, and Tourette syndrome, excluded. We also excluded hoarding disorder that belongs to the category of Obsessive-Compulsive and Related Disorders. Two investigators manually reviewed all potential studies (i.e., study title, conditions and, if necessary, the full ClinicalTrials.gov records) to determine relevance to OCD.

### Data Collection

The following data from each eligible trial were independently extracted by two investigators: country, general characteristics, design characteristics, interventions, study status, results reporting, location of study procedures, collaborators, and funding source. We also searched PubMed using the ClinicalTrials.gov identifier of each trial to identify related publications. Publication of the results within 12 months from study completion was regarded as timely publication; otherwise, it was considered delayed publication. The duration of the study was calculated from the initiation of the investigation to study completion. For studies with missing data regarding the date of completion and the date of last update posted was utilized. For ongoing studies, study duration was calculated from the initiation of the investigation to May 5, 2020. For trials with more than one sponsor, only data regarding the primary sponsor were extracted. Interventions are categorized as behavioral, drug, device/procedure, dietary supplement, and radiation.

### Statistical Analysis

Categorical variables were presented as frequencies and percentages. A survival analysis was performed to determine the time from study report and weighted cumulative publication rate for all completed studies. Early trial discontinuation was defined as a study status including suspended, terminated, or withdrawn. Univariate and multivariate Cox analyses were performed to investigate factors associated with early trial discontinuation. The hazard ratio (HR) and 95% confidence interval (CI) were reported. The regression analysis was restricted to interventional trials initiated between 2000 and May 7, 2020. After performing the regression analysis, we carried out a survival analysis to present the duration of trials and weighted cumulative rate of early trial discontinuation based on a subgroup of significant factors. A sensitivity analysis, excluding trials that involved only children, was conducted. Univariate and multivariate logistic regression analyses were used to investigate factors associated with reporting results in registry and publication after completion, including the intervention type, collaborators, phase, funding source, allocation, blinding, primary purpose, and enrollment. For the statistical analysis, the significance level was set at 0.05, and the analysis was performed using the SPSS version 18.0 (SPSS Inc., Chicago, IL, USA) software.

## Results

### General Characteristics

We retrieved 433 trials; based on the inclusion/exclusion criteria, 298 trials were finally selected for the analysis. The ClinicalTrials.gov identifiers of the included OCD trials are shown in Supplementary Table 1. The general characteristics of the identified trials are shown in Table 1. In terms of the number of OCD trials, this study demonstrated an increasing trend after 2000, peaking in 2014, and followed by a decreasing trend in 2015–2018. Trials that included only OCD and OCD combined with other diseases accounted for 90.3 and 9.7% of the selected studies, respectively. Most trials included both males and females (*n* = 296, 99.3%). Among the OCD trials, 78.5% included only adult patients, while 15.1% included only pediatric patients. Between 2011 and 2020, 68.5% of the OCD trials were carried out. Only 10.7% of the trials were completed within 1 year. The majority of trials required 1–3 and 3–5 years for completion (37.9 and 26.2%, respectively). Small studies comprised 84.2% of OCD trials, involving <100 patients.

**Table 1:** General characteristic of OCD trials registered in ClinicalTrials.gov (*n* = 298).

**Items**	**Detail**	**Number**	**Percent**
Conditions	OCD	269	90.3
	OCD+others	29	9.7
Gender	All	296	99.3
	Female	2	0.7
Age	Adult	234	78.5
	Child	45	15.1
	Child and adult	19	6.3
Start date	Prior to 2000	8	2.7
	2001–2010	93	31.2
	2011–2020	196	65.8
	Not provided	1	0.3
During date	0–12 months	32	10.7
	13–24 months	51	17.1
	25–36 months	62	20.8
	37–48 months	51	17.1
	49–60 months	27	9.1
	60 months	64	21.5
	Not provided	11	3.7
Enrollment	0–100	251	84.2
	101–200	36	12.1
	200–	6	2.0
	Not provided	5	1.7
Country	United States	146	49.0
	Canada	27	9.1
	China	18	6.0
	Brazil	15	5.0
	France	15	5.0
	Sweden	15	5.0
	Israel	12	4.0
	Germany	10	3.4
	Others	40	13.4
Location of study procedures	University/hospital	178	59.7
	Other	95	31.9
	Industry	18	6.0
	NIH	7	2.3
Collaborators	1	83	27.9
	2	145	48.7
	3	42	14.1
	4	16	5.4
	5	9	3.0
	6	3	1.0
Funded by^*^	Other	277	93.0
	NIH	43	14.4
	Industry	39	13.1
Status	Completed	155	52.0
	Ongoing	88	29.5
	Stopped early	28	9.4
	Unknown status	27	9.1
Study results for completed trials	Has results	37	76.1
	Submit the results to the registry within 1 year of the primary completion date	5	3.2
Publications	Yes	62	20.8
	1	45	15.1
	2	9	3.0
	3	4	1.3
	4	4	1.3
	No	236	79.2
Time from completion to publication	≤ 1 year	14	29.2
	>1 year	34	71.8

*OCD, obsessive-compulsive disorder; NIH, National Institutes of Health*.

* *Some included trials have more than one funding resources*.

A total of 24 countries have registered OCD trials in ClinicalTrials.gov. The USA registered approximately half of the trials (49%), followed by Canada (9.1%), China (6%), and Brazil (5%). Seven countries registered >10 trials.

Most of the locations of study procedures of OCD trials were universities/hospitals, accounting for nearly 60% of the studies, followed by industries (6%) and the National Institutes of Health (NIH; Bethesda, MD, USA) (2.3%) (Table 1). Of OCD trials, 72.1% involved more than one collaborator. Trials with two collaborators accounted for the greatest proportion (nearly 50%). In terms of funding, only 14.4 and 13.1% of the OCD trials were funded by the NIH and industries, respectively.

Of the 298 identified OCD trials, 28 trials (nearly 10%) were discontinued early, including two suspended, 17 terminated, and nine withdrawn trials (Table 1). A total of 155 trials (52%) were completed; however, 118 trials (76.1%) did not submit their results to the registry. Only five trials submitted their results to the registry within 1 year from the primary completion date. The survival analysis of publications is shown in Supplementary Figure 1. A total of 48 trials were published after completion. Nevertheless, >70% of those were published after 12 months (median time: 19.5 months). The longest time required for the publication of a trial was 104 months.

### Interventions in Obsessive-Compulsive Disorder Trials

The treatment-aimed trials involved five categories; the most common type of intervention was behavioral therapy (39.0%) (Supplementary Table 2). There were 20 categories for behavioral therapy, among which cognitive behavioral therapy was the most common (59.2%). Drug intervention accounted for 35.1% of studies, involving 47 categories and 30 types of drugs. Device/procedure intervention accounted for 24.7% of studies, involving 12 categories; the most common types were deep brain stimulation (38.7%) and repetitive transcranial magnetic stimulation (33.9%).

### Design Characteristics

Table 2 summarizes the design characteristics of the selected OCD trials. Of the 298 trials, less than half provided information regarding their phase, as most of them were non-drug trials. Of the trials, 33.6% were Phases 1–3 trials. Of the OCD studies, 67.8% were randomized. In trials including controlled groups (72.2%), the vast majority (82.3%) performed parallel assessment. Notably, 61.4% of the studies were blind trials (single- or double-blind trials, accounting for 18.5% each). Of the 183 trials that utilized blinding, 176 trials reported blinded objects. The most common blinded object was outcome assessor (*n* = 132), followed by investigator (*n* = 115), participant (*n* = 101), and care provider (*n* = 57). The primary purpose of the OCD trials was treatment (84.2%).

**Table 2:** Design characteristic of OCD trials registered in ClinicalTrials.gov (n = 298).

**Items**	**Detail**	**Number**	**Percent**
Phases	Early phase 1	2	0.7
	Phase 1	10	3.4
	Phase 1|Phase 2	9	3.0
	Phase 2	55	18.5
	Phase 2|Phase 3	6	2.0
	Phase 3	18	6.0
	Phase 4	34	11.4
	Not provided	164	55.0
Allocation	Randomized	202	67.8
	Non-randomized	28	9.4
	Not provided	68	22.8
Intervention model	Parallel assignment	177	59.4
	Crossover assignment	30	10.1
	Factorial assignment	6	2.0
	Sequential assignment	2	0.7
	Single group assignment	77	25.8
	Not provided	6	2.0
Masking	Single	55	18.5
	Double	55	18.5
	Triple	28	9.4
	Quadruple	45	15.1
	None (open label)	110	36.9
	Not provided	5	1.7
Primary purpose	Treatment	251	84.2
	Basic science	13	4.4
	Diagnostic	4	1.3
	Supportive care	2	0.7
	Device feasibility	1	0.3
	Educational/counseling/training	1	0.3
	Health services research	1	0.3
	Prevention	1	0.3
	Other	16	5.4
	Not provided	8	2.7

*OCD, obsessive-compulsive disorder*.

### Characteristics Associated With Early Trial Discontinuation

Reasons provided for withdrawn, terminated, and suspended trials included recruitment problems (*n* = 9), funding/resource problems (*n* = 9), departure of the principal investigator from the institution (*n* = 4), lack of efficacy (*n* = 3), study design problems (*n* = 1), administrative reason (*n* = 1), and interim analysis results (*n* = 1). Univariate Cox regression analysis revealed a significant difference in the rate of early trial discontinuation between the different types of interventions, involvement or absence of collaborators, and different funding sources (Table 3). Drug trials (HR = 2.56, 95% CI: 1.21–5.43), absence of collaborators (HR = 3.87, 95% CI: 1.62–9.26), and industry trials (HR = 3.97, 95% CI: 1.49–10.53) were more frequently discontinued early compared with non-drug trials, trials with collaborators, and NIH/other trials. Considering the significant association between the involvement or absence of collaborators and funding sources, further multivariate Cox regression analysis was performed. A significant collinearity was found between the variables “interventions” and “source of funding” when simultaneously included in the regression model. We only excluded the variable “source of funding” in our multivariate analysis. The results showed that drug trials (HR = 3.93, 95% CI: 1.70–9.08) and absence of collaborators (HR = 5.17, 95% CI: 1.97–13.54) were independent risk factors for early trial discontinuation. The sensitivity analysis yielded consistent results (Supplementary Table 3).

**Table 3:** Cox regression analysis of OCD trials characteristics associated with early trial discontinuation.

**Variable**		**Univariate**		**Multivariate**	
		**HR (95% CI)**	***P***	**HR (95% CI)**	***P***
Intervention	Non-drug	-	-		
	Drug	2.563 (1.210–5.425)	0.014	3.925 (1.697–9.078)	0.001
Collaborators	Yes	-	-		
	No	3.872 (1.620–9.255)	0.002	5.169 (1.974–13.535)	0.001
Phase	Phase 4 or not provided	-	-		
	Phase 1–3	1.036 (0.477–2.254)	0.928		
Enrollment	0–100				
	>100	5.423 (0.734–40.046)	0.097		
Funded by	NIH/others	-	-		
	Industry	3.966 (1.494–10.527)	0.006		
Allocation	Non-randomized	-	-		
	Randomized	0.743 (0.174–3.168)	0.688		
Blinding	Non	-	-		
	Single	0.532 (0.115–2.466)	0.688		
	Double-quadruple	1.546 (0.688–3.476)	0.292		
Primary purpose	Non-treatment				
	Treatment	1.042 (0.311–3.489)	0.946		

*OCD, obsessive-compulsive disorder; HR, hazard ratio; NIH, National Institutes of Health*.

### Characteristics Associated With Reporting Results in the Registry

Univariate logistic regression showed significant differences in the rate of reporting results in the registry, according to different types of interventions and blinding. Premarket trials were significantly different from post-market trials (Table 4). Drug trials (OR = 7.14, 95% CI: 3.04–16.7), premarket trials (OR = 5.91, 95% CI: 2.61–13.36), and double-quadruple trials (OR = 7.70, 95% CI: 1.67–35.42) were more frequently reported results than non-drug trials, post-market trials, and non-blinded trials. Further multivariate logistic regression shows that non-drug trials were less likely to report results in ClinicalTrials.gov than were drug trials (OR = 3.32, 95% CI: 1.21–9.11). Absence of collaborators (OR = 3.25, 95% CI: 1.10–9.60) was an independent risk factors for unreported results. Double-quadruple trials (OR = 5.23, 95% CI: 1.05–26.2) more frequently reported results than non-blind trials.

**Table 4:** Logistic regression analysis of OCD trials characteristics associated with study results for completed trials.

**Variable**		**Univariate**		**Multivariate**	
		**OR (95% CI)**	***P***	**OR (95% CI)**	***P***
Intervention	Non-drug	Ref		Ref	
	Drug	7.137 (3.044–16.736)	<0.001	3.317 (1.208–9.111)	0.020
Collaborators	No	Ref		Ref	
	Yes	2.130 (0.986–4.602)	0.054	3.251 (1.102–9.596)	0.033
Phase	Phase 4 or not provided	Ref			
	Phase 1–3	5.909 (2.614–13.356)	<0.001		
Enrollment	0–100	Ref			
	>100	0.599 (0.162–2.212)	0.442		
Funded by	Industry	Ref			
	NIH/others	2.400 (0.285–20.180)	0.420		
Allocation	Non-randomized	Ref			
	Randomized	1.975 (0.414–9.408)	1.975		
Blinding	Non	Ref		Ref	
	Single	1.819 (0.810–4.088)	0.147	3.416 (0.967–12.063)	0.056
	Double-quadruple	7.700 (1.674–35.417)	0.009	5.231 (1.045–26.193)	0.044
Primary Purpose	Non-treatment	Ref			
	Treatment	1.753 (0.365–8.404)	0.483		

*OCD, obsessive-compulsive disorder; NIH, National Institutes of Health*.

### Characteristics Associated With Publications for Completed Trials

Univariate and multivariate logistic regression analyses of OCD trials failed to reveal any trial characteristics that were significantly associated with completed trial publication (Supplementary Table 4).

## Discussion

The purpose of this analysis was to help researchers better understand changes in the key characteristics of OCD trials over time. The number of registered OCD trials declined after 2014. The trend of decreasing quantity was also observed in the mental health field (9). This may partly explain the decrease in the number of OCD trials registered by the USA in recent years.

The classification of OCD was updated in 2013. According to the classification criteria of the *Diagnostic and Statistical Manual of Mental Disorders*, 5th edition (DSM-5), hoarding disorders trials that were labeled as OCD in ClinicalTrials.gov were excluded. The sample sizes of OCD trials were generally small, with the majority (84.2%) enrolling ≤ 100 participants. Typically, these investigations could not provide sufficient evidence for the development of guidelines concerning OCD. As stated earlier, the primary purpose of the majority (>84%) of OCD trials was treatment. Only ~4% of the trials belonged to the category of basic science. Moreover, very few trials belonged to the category of prevention, supportive care, and diagnosis. In the treatment category, the most common types of trials were behavioral treatment trials (~40%), followed by drug treatment trials (35%) and device/procedure treatment trials (25%). Among the 98 behavioral treatment trials identified, our analysis showed that cognitive behavioral therapy accounted for 60% of studies. When evaluating 62 device/procedure trials, this analysis identified 24 and 21 trials focused on deep brain stimulation and repetitive transcranial magnetic stimulation, respectively.

The present results showed that 9.4% of OCD trials were discontinued early. This rate was slightly higher than that reported by Arnow et al. (8.4%) (10). Recruitment and funding/resource problems were the most common reasons of discontinuation, which have been previously reported as the major cause of trial discontinuation (11). Multivariate logistic regression analysis showed that drug intervention (HR = 3.925, 95% CI: 1.697–9.078) and non-collaboration (HR = 5.169, 95% CI: 1.974–13.535) trials registered in ClinicalTrials.gov were more like to be discontinued prior to completion. Funding of drug trials by manufacturers could explain the collinearity between the variables “interventions” and “source of funding.” Non-collaboration trials were more like to be discontinued early than collaboration trials, which could be related to difficulty in recruitment. In addition, drug intervention trials were more likely to be discontinued early than non-drug intervention trials. This could be related to the funding of most drug trials in the OCD field by industries, which may have less tolerance for risk and a shorter view of return on investment (12). Drug trials and premarket trials more frequently provided results in registry, which may be related to premarket approval.

Underreporting and delayed reporting remain important problems in the OCD field. The present findings revealed that 76.1% of completed trials did not report results to the registry. This percentage was markedly higher than the non-reporting rate previously recorded by Wortzel et al. (57.7%) (9). Early-phase trials were significantly less likely to report results. The Food and Drug Administration Amendments Act and Final Rule only mandated the reporting of results for pharmaceutical or device trials (13). However, of the 298 OCD trials included in this study, only 150 trials (50.3%) were pharmaceutical or device trials. Furthermore, only 87 pharmaceutical or device trials (29.2%) were registered by institutes in the USA, potentially explaining the lower rate of reporting in the OCD field. Timely reporting and publication of results to the registry are very important. The bias caused by delayed and selective reporting contributes to the wasting of research resources (14) and impairs clinical practice, which may cause harm to patients. Therefore, underreporting and delayed reporting of results fail to fulfill the ethical obligation to subjects participating in trials (15).

The present study showed that only 13.1% of the OCT trials were funded by industry. This percentage was lower than that reported by Wortzel et al. in OCD trials (22/131, 16.8%) in the USA (9). Wortzel et al. also reported that mental health trials were less frequently funded by industry than non-mental health trials (17.6 vs. 39.7%, respectively). Furthermore, a decreasing trend was found in industry-funded mental health trials. The main reasons for the reduced investment by international pharmaceutical companies in the discovery of new drugs for mental health disorders included the increased cost of R&D, long time required to launch a drug to the market, reduced market exclusivity for new drugs, and the decrease in demand for branded drugs by increasingly cost-conscious payers.

This study showed that fewer OCD trials were randomized than overall mental health trials (67.8 vs. 79.9%, respectively). A lower proportion of randomization could be explained by the fact that more than one-quarter (25.8%) of the OCD trials implemented a signal arm design. Of note, more OCD trials utilized blinding than overall mental health trials (61.4 vs. 58.5%, respectively). In the previous 5 years, there was a decreasing trend in the proportion of trials utilizing blinding (from 67.7% in 2011–2015 to 59.8% in 2016–2020). This decrease could be explained by the increased number of behavioral intervention trials, many of which cannot be blinded.

This study had several limitations. Firstly, this study was focused on the methodological features of OCD trials; therefore, the relevance of its findings to the clinical field was not analyzed. Secondly, we only searched the ClinicalTrials.gov database, in which only approximately 50% of trials were registered with limited registration information. This analysis did not include published but unregistered OCD trials, comorbidity studies, and trials conducted outside of the US Food and Drug Administration jurisdiction. Hence, the conclusions of this study should be interpreted with caution. Nonetheless, information submitted to ClinicalTrials.gov is relatively reliable due to the strict quality control review conducted by members of the National Library of Medicine (16). Analysis of early discontinuation of clinical trials in other specialties adopted the same approach (17).

Thirdly, we only searched the title and abstract fields in PubMed using National Clinical Trial numbers to analyze the publication status of registered OCD trials. Some non-PubMed Central indexed publications could be missed if their National Clinical Trial numbers are only reported in the full-text articles. Moreover, publications only indexed in other databases, such as Embase, could have been missed. Fourthly, among the 298 OCD trials included in this analysis, only 28 trials were discontinued early; therefore, the sample size could be insufficient for the Cox regression analysis on risk factors associated with discontinuation. Finally, there were some missing data on “Completion Date”; in such cases, the “Last Update Posted” was used to fill these missing data. Hence, the correlative factors should be interpreted with caution due to the small number of trials that were discontinued early (*n* = 28).

In conclusion, there was an increasing trend in the number of OCD trials registered in ClinicalTrials.gov. Nearly 50% of these trials were conducted in the USA. The majority of these trials involved <200 participants. The primary purpose of 84% of the OCD trials was treatment, and behavioral therapy was the most frequently used intervention. Few OCD trials were funded by pharmaceutical companies. Discontinuation and delayed publication were problems of OCD trials. Funding by pharmaceutical companies and absence of collaborators were individual risk factors for early trial discontinuation. Future investigation is warranted to strengthen the design, implementation, and timely and transparent reporting of OCD trials.

## Data Availability Statement

The original contributions presented in the study are included in the article/Supplementary Material, further inquiries can be directed to the corresponding author/s.

## Author Contributions

MC and GL contributed to the conception of the study. XW, LL, ZY, TH, and LD performed trials search and selection. SL, QG, LY, YC, and YL performed data compilation and statistical analysis. SL, QG, LY, and YL wrote the manuscript. MC and GL helped perform the analysis with constructive discussions. All authors contributed to the article and approved the submitted version.

## Conflict of Interest

The authors declare that the research was conducted in the absence of any commercial or financial relationships that could be construed as a potential conflict of interest.

## Publisher's Note

All claims expressed in this article are solely those of the authors and do not necessarily represent those of their affiliated organizations, or those of the publisher, the editors and the reviewers. Any product that may be evaluated in this article, or claim that may be made by its manufacturer, is not guaranteed or endorsed by the publisher.
