# Supplementary figures and images for: Characteristic and Early Discontinuation of Obsessive-Compulsive Disorder Trials Registered on ClinicalTrials.gov
Source: Front Psychiatry. 2021 Jul 26;12:650057. doi: 10.3389/fpsyt.2021.650057 (PMC8350037; doi:10.3389/fpsyt.2021.650057)

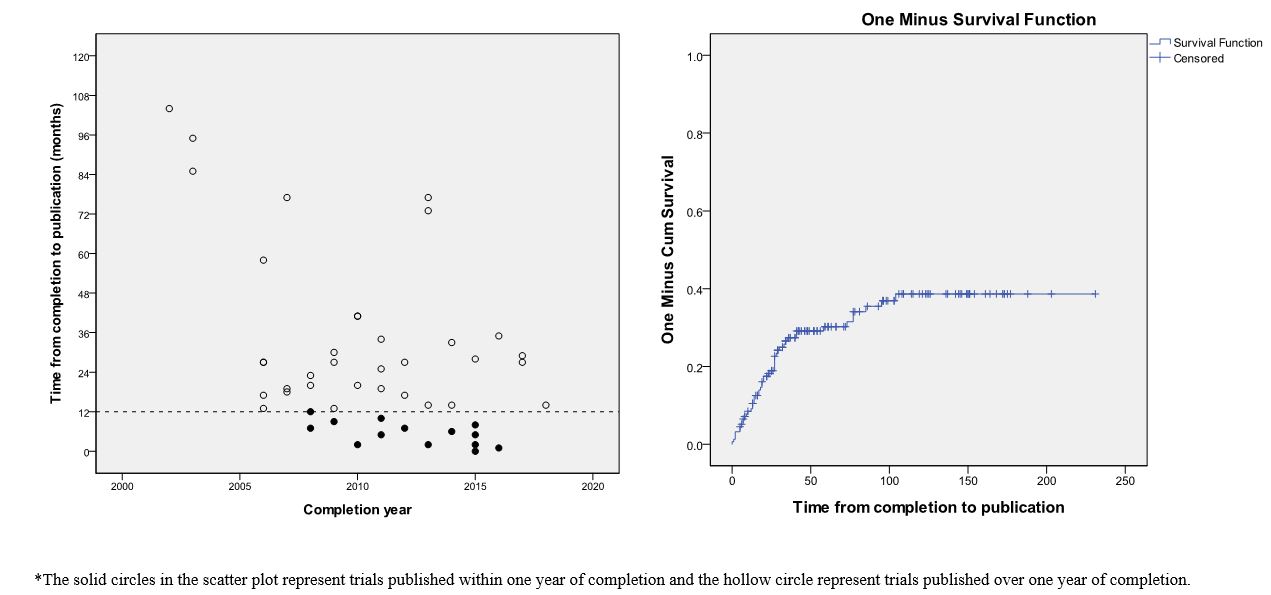

Supplement: Supplementary file 1 [file Figure_1.JPEG]
