# Supplementary material for: Characteristic and Early Discontinuation of Obsessive-Compulsive Disorder Trials Registered on ClinicalTrials.gov
Source: Front Psychiatry. 2021 Jul 26;12:650057. doi: 10.3389/fpsyt.2021.650057 (PMC8350037; doi:10.3389/fpsyt.2021.650057)
Supplement: Supplementary file 2 [file Data_Sheet_1.docx]

Appendix Table-1 ClinicalTrials.gov Identifier (NCT number) of included OCD trials

| **NCT Number** | | | | |
| --- | --- | --- | --- | --- |
| NCT00000373 | NCT00389493 | NCT01043900 | NCT01807403 | NCT02329587 |
| NCT00000384 | NCT00396552 | NCT01061983 | NCT01695291 | NCT02334644 |
| NCT00000386 | NCT00399022 | NCT01093976 | NCT01708226 | NCT02355002 |
| NCT00001359 | NCT00405535 | NCT01100255 | NCT01794156 | NCT02375152 |
| NCT00001658 | NCT00456937 | NCT01108393 | NCT01809990 | NCT02377375 |
| NCT00004310 | NCT00464698 | NCT01135745 | NCT01813019 | NCT02378896 |
| NCT00035438 | NCT00466609 | NCT01148316 | NCT01833442 | NCT02398045 |
| NCT00045903 | NCT00471588 | NCT01172275 | NCT01849809 | NCT02398318 |
| NCT00055068 | NCT00515255 | NCT01172873 | NCT01933919 | NCT02407288 |
| NCT00057603 | NCT00523718 | NCT01194076 | NCT01949753 | NCT02421315 |
| NCT00073346 | NCT00533806 | NCT01242735 | NCT01953042 | NCT02422290 |
| NCT00074815 | NCT00539513 | NCT01275248 | NCT01981317 | NCT02431845 |
| NCT00106249 | NCT00564564 | NCT01281969 | NCT01985815 | NCT02433886 |
| NCT00116532 | NCT00592852 | NCT01303536 | NCT01986296 | NCT02437773 |
| NCT00126282 | NCT00616486 | NCT01329133 | NCT02006199 | NCT02450695 |
| NCT00169377 | NCT00632229 | NCT01331876 | NCT02014194 | NCT02467374 |
| NCT00182000 | NCT00640133 | NCT01343732 | NCT02018185 | NCT02500888 |
| NCT00182520 | NCT00674219 | NCT01347099 | NCT02018848 | NCT02511392 |
| NCT00184262 | NCT00680602 | NCT01348529 | NCT02022709 | NCT02528331 |
| NCT00187928 | NCT00690729 | NCT01349231 | NCT02059980 | NCT02541812 |
| NCT00211744 | NCT00708240 | NCT01368510 | NCT02062658 | NCT02541968 |
| NCT00215137 | NCT00708396 | NCT01371110 | NCT02098148 | NCT02590445 |
| NCT00216294 | NCT00723060 | NCT01404208 | NCT02103621 | NCT02601677 |
| NCT00229320 | NCT00724490 | NCT01404871 | NCT02114918 | NCT02602886 |
| NCT00229385 | NCT00728923 | NCT01409642 | NCT02136953 | NCT02616341 |
| NCT00234689 | NCT00742664 | NCT01411774 | NCT02160431 | NCT02624596 |
| NCT00251303 | NCT00743834 | NCT01414023 | NCT02191631 | NCT02635178 |
| NCT00254735 | NCT00748761 | NCT01447966 | NCT02194075 | NCT02655926 |
| NCT00257361 | NCT00758966 | NCT01483339 | NCT02196090 | NCT02656342 |
| NCT00264238 | NCT00796497 | NCT01522287 | NCT02202915 | NCT02656784 |
| NCT00299611 | NCT00822601 | NCT01525576 | NCT02206776 | NCT02660099 |
| NCT00305500 | NCT00854919 | NCT01540305 | NCT02206945 | NCT02663167 |
| NCT00316316 | NCT00864123 | NCT01555970 | NCT02216981 | NCT02671266 |
| NCT00316355 | NCT00881465 | NCT01596608 | NCT02217995 | NCT02685280 |
| NCT00318539 | NCT00932204 | NCT01617083 | NCT02221518 | NCT02704117 |
| NCT00352768 | NCT00956085 | NCT01635569 | NCT02229903 | NCT02713516 |
| NCT00358995 | NCT00994786 | NCT01649895 | NCT02234011 | NCT02739061 |
| NCT00363298 | NCT00997087 | NCT01659125 | NCT02253472 | NCT02743715 |
| NCT00369642 | NCT01004302 | NCT01674361 | NCT02267629 | NCT02773082 |
| NCT00374348 | NCT01031927 | NCT01686087 | NCT02285699 | NCT02790710 |
| NCT00382291 | NCT01035242 | NCT01687140 | NCT02314195 | NCT02797808 |
| NCT02818088 | NCT03207633 | NCT03420495 | NCT03799419 | NCT04086446 |
| NCT02844049 | NCT03211221 | NCT03451409 | NCT03826693 | NCT04106102 |
| NCT02850393 | NCT03212703 | NCT03457675 | NCT03852186 | NCT04131829 |
| NCT02867449 | NCT03217123 | NCT03463590 | NCT03855943 | NCT04136626 |
| NCT02884674 | NCT03239210 | NCT03473080 | NCT03856567 | NCT04154085 |
| NCT02886780 | NCT03241056 | NCT03476902 | NCT03874611 | NCT04165577 |
| NCT02904356 | NCT03263546 | NCT03496285 | NCT03894085 | NCT04217408 |
| NCT02909660 | NCT03265015 | NCT03511534 | NCT03894397 | NCT04228744 |
| NCT02911324 | NCT03273699 | NCT03572543 | NCT03918577 | NCT04281134 |
| NCT02926365 | NCT03274440 | NCT03584945 | NCT03918837 | NCT04286126 |
| NCT02955654 | NCT03284671 | NCT03595098 | NCT03921255 | NCT04313439 |
| NCT02973932 | NCT03299166 | NCT03601312 | NCT03926546 | NCT04323566 |
| NCT03002753 | NCT03300947 | NCT03649685 | NCT03929081 | NCT04336228 |
| NCT03068429 | NCT03304600 | NCT03661905 | NCT03956771 | NCT03184454 |
| NCT03128749 | NCT03343106 | NCT03667807 | NCT03983031 | NCT03184454 |
| NCT03156335 | NCT03348930 | NCT03672565 | NCT03993535 | NCT03184454 |
| NCT03172728 | NCT03356483 | NCT03677947 | NCT04015596 | NCT03184454 |
| NCT03179839 | NCT03393078 | NCT03682913 | NCT04042038 | NCT03182075 |
| NCT03404609 | NCT03754647 | NCT04071990 |  |  |

Appendix Table-2 Interventions of OCD trials registered in ClinicalTrials.gov aimed at treatment

| Category | Interventions | Number | Percent |  | Category | Interventions | Number | Percent |
| --- | --- | --- | --- | --- | --- | --- | --- | --- |
| Behavioral | Cognitive Behavior Therapy | 58 | 59.2 |  |  | Bitopertin | 1 | 1.1 |
|  | Exposure and Response Prevention | 10 | 10.2 |  |  | Dextro-amphetamine | 1 | 1.1 |
|  | Attention Modification Program | 4 | 4.1 |  |  | Dronabinol | 1 | 1.1 |
|  | Mindfulness | 4 | 4.1 |  |  | Flumazenil | 1 | 1.1 |
|  | Acceptance and Commitment Therapy | 2 | 2.0 |  |  | Gamunex Intravenous Immunoglobulin | 1 | 1.1 |
|  | Exercise | 2 | 2.0 |  |  | Luvox CR | 1 | 1.1 |
|  | Neurofeedback | 2 | 2.0 |  |  | Marijuana | 1 | 1.1 |
|  | Personalized Computer Program | 2 | 2.0 |  |  | Nabilone | 1 | 1.1 |
|  | Psycho-education pamphlet | 2 | 2.0 |  |  | Naproxen Sodium | 1 | 1.1 |
|  | Psychotherapy | 2 | 2.0 |  |  | Nitrous Oxide | 1 | 1.1 |
|  | Association splitting | 1 | 1.0 |  |  | NPL-2003 | 1 | 1.1 |
|  | Booster | 1 | 1.0 |  |  | Olanzapine | 1 | 1.1 |
|  | Concentrated exposure treatment (cET) | 1 | 1.0 |  |  | Paliperidone | 1 | 1.1 |
|  | Gradual Exposure (EXP-G) | 1 | 1.0 |  |  | Paroxetine | 1 | 1.1 |
|  | Inference Based Approach (IBA) | 1 | 1.0 |  |  | Penicillin | 1 | 1.1 |
|  | Kundalini Yoga Meditation | 1 | 1.0 |  |  | Pramipexole, Amisulpride | 1 | 1.1 |
|  | Motivational interviewing | 1 | 1.0 |  |  | Pregabalin | 1 | 1.1 |
|  | OCFighter | 1 | 1.0 |  |  | Rapastinel | 1 | 1.1 |
|  | Personalized Inhibitory Control Training protocol | 1 | 1.0 |  |  | Risperidone | 1 | 1.1 |
|  | Therapist assisted mobile intervention | 1 | 1.0 |  |  | Seromycin | 1 | 1.1 |
|  | **SubTotal** | **98** |  |  |  | Sertraline | 1 | 1.1 |
| Drug | Ketamine | 7 | 8.0 |  |  | St. John’s Wort | 1 | 1.1 |
|  | Escitalopram | 6 | 6.8 |  |  | Tolcapone | 1 | 1.1 |
|  | D-Cycloserine | 5 | 5.7 |  |  | Xenon | 1 | 1.1 |
|  | Memantine | 5 | 5.7 |  |  | **SubTotal** | **88** |  |
|  | Fluoxetine | 4 | 4.5 |  | Device/Procedure | Deep brain stimulation | 24 | 38.7 |
|  | Ondansetron | 4 | 4.5 |  |  | Refractory transcranial magnetic stimulation (rTMS) | 21 | 33.9 |
|  | Fluvoxamine | 3 | 3.4 |  |  | Transcranial direct current stimulation | 4 | 6.5 |
|  | N-Acetylcysteine | 3 | 3.4 |  |  | Gamma Ventral Capsulotomy | 3 | 4.8 |
|  | Quetiapine | 3 | 3.4 |  |  | Magnetic Seizure Therapy | 2 | 3.2 |
|  | SSRIs | 3 | 3.4 |  |  | Smartphone-delivered cognitive behavioral therapy | 2 | 3.2 |
|  | Topiramate | 3 | 3.4 |  |  | Anterior Capsulotomy | 1 | 1.6 |
|  | Clomipramine | 2 | 2.3 |  |  | Deep Brain Stimulation | 1 | 1.6 |
|  | Duloxetine | 2 | 2.3 |  |  | Deep Transcranial Magnetci Stimulation | 1 | 1.6 |
|  | Glycine | 2 | 2.3 |  |  | ExAblate Transcranial System | 1 | 1.6 |
|  | Psilocybin | 2 | 2.3 |  |  | Micro-assisted technique | 1 | 1.6 |
|  | Riluzole | 2 | 2.3 |  |  | Mindfulness | 1 | 1.6 |
|  | Rituximab | 2 | 2.3 |  |  | **SubTotal** | **62** |  |
|  | AFQ056 | 1 | 1.1 |  | Dietary Supplement | Probiotic Formula | 1 | 50.0 |
|  | Agomelatine A | 1 | 1.1 |  |  | Vitamin C | 1 | 50.0 |
|  | Amoxicillin | 1 | 1.1 |  |  | **SubTotal** | **2** |  |
|  | Aripiprazole | 1 | 1.1 |  | Radiation | Radiosurgery | 1 |  |
|  | Azithromycin | 1 | 1.1 |  |  | SubTotal | 1 |  |
|  | BHV-4157 | 1 | 1.1 |  |  | **Total** | 251 |  |

Appendix Table-3 Cox regression analysis of OCD trials characteristics associated with early trial discontinuation (sensitive analysis)

| Variable |  | Univariate | | Multivariate | |
| --- | --- | --- | --- | --- | --- |
|  |  | HR (95%CI) | P | HR (95%CI) | P |
| Intervention | Non-drug | - | - |  |  |
|  | Drug | 2.538(1.149-5.605) | 0.021 | 3.800 (1.596-9.048) | 0.003 |
| Collaborators | Yes | - | - |  |  |
|  | No | 3.167(1.291-7.768) | 0.012 | 4.169 (1.563-11.119) | 0.004 |
| Phase | Phase 4 or Not provided | - | - |  |  |
|  | Phase 1-3 | 1.344 (0.601-3.006) | 0.471 |  |  |
| Enrollment |  |  |  |  |  |
|  |  | 4.209 (0.567-31.255) | 0.160 |  |  |
| Funded Bys | NIH/Others | - | - |  |  |
|  | Industry | 3.010 (1.023-8.855) | 0.045 |  |  |
| Allocation | Non- Randomized | - | - |  |  |
|  | Randomized | 0.042 (0.000-15.563) | 0.293 |  |  |
| Blinding | Non | - | - |  |  |
|  | Single | 0.888 (0.178-4.428) | 0.885 |  |  |
|  | Double-Quadruple | 2.185 (0.859-5.556) | 0.101 |  |  |
| Primary Purpose | Non-treatment |  |  |  |  |
|  | Treatment | 0.904 (0.267-3.062) | 0.872 |  |  |

Appendix Table-4 Logistic regression analysis of OCD trials characteristics associated with publications for completed trials

| Variable |  | Univariate | | | Multivariate | |
| --- | --- | --- | --- | --- | --- | --- |
|  |  | OR (95%CI) | P | | OR (95%CI) | P |
| Intervention | Non-drug | Ref |  | |  |  |
|  | Drug | 1.774 (0.888-3.542) | 0.104 | |  |  |
| Collaborators | No | Ref |  | |  |  |
|  | Yes | 1.635 (0.819-3.266) | 0.164 | |  |  |
| Phase | Phase 4 or Not provided | Ref |  | |  |  |
|  | Phase 1-3 | 1.449 (0.723-2.904) | 0.296 | |  |  |
| Enrollment | 0-100 | Ref |  |  | |  |
|  | >100 | 1.130 (0.391-3.264) | 0.821 | |  |  |
| Funded Bys | Industry | Ref |  | |  |  |
|  | NIH/Others | 3.613 (0.432-30.222) | 0.236 | |  |  |
| Allocation | Non- Randomized | Ref |  | |  |  |
|  | Randomized | 1.111 (0.346-3.566) | 0.859 | |  |  |
| Blinding | Non | Ref |  | |  |  |
|  | Single | 0.834 (0.310-2.244) | 0.720 | |  |  |
|  | Double-Quadruple | 1.353 (0.628-2.917) | 0.441 | |  |  |
| Primary Purpose | Non-treatment | Ref |  | |  |  |
|  | Treatment | 1.023 (0.292-3.587) | 0.971 | |  |  |
